# Assessment of the In Vitro Phosphatidylinositol Glycan Class A (PIG-A) Gene Mutation Assay Using Human TK6 and Mouse Hepa1c1c7 Cell Lines

**DOI:** 10.3390/jox14030073

**Published:** 2024-09-19

**Authors:** Wenhao Zhang, Charles A. Miller, Mark J. Wilson

**Affiliations:** 1 Department of Environmental Health Sciences, School of Public Health and Tropical Medicine, Tulane University, New Orleans, LA 70112, USA; rellim@tulane.edu (C.A.M.); mark.wilson@ul.org (M.J.W.); 2 Chemical Insights Research Institute of Underwriters Laboratories Research Institutes, Marietta, GA 30067, USA

**Keywords:** gene mutation, in vitro PIG-A gene mutation assay, ethyl methane sulfonate (EMS), polycyclic aromatic hydrocarbons (PAHs)

## Abstract

Gene mutations linked to diseases like cancer may be caused by exposure to environmental chemicals. The X-linked phosphatidylinositol glycan class A (PIG-A) gene, required for glycosylphosphatidylinositol (GPI) anchor biosynthesis, is a key target locus for in vitro genetic toxicity assays. Various organisms and cell lines may respond differently to genotoxic agents. Here, we compared the mutagenic potential of directly genotoxic ethyl methane sulfonate (EMS) to metabolically activated pro-mutagenic polycyclic aromatic hydrocarbons (PAHs). The two classes of mutagens were compared in an in vitro PIG-A gene mutation test using the metabolically active murine hepatoma Hepa1c1c7 cell line and the human TK6 cell line, which has limited metabolic capability. Determination of cell viability is required for quantifying mutagenicity. Two common cell viability tests, the MTT assay and propidium iodide (PI) staining measured by flow cytometry, were evaluated. The MTT assay overestimated cell viability in adherent cells at high benzo[a]pyrene (B[a]P) exposure concentrations, so PI-based cytotoxicity was used in calculations. The spontaneous mutation rates for TK6 and Hepa1c1c7 cells were 1.87 and 1.57 per million cells per cell cycle, respectively. TK6 cells exposed to 600 µM and 800 µM EMS showed significantly higher mutation frequencies (36 and 47 per million cells per cell cycle, respectively). Exposure to the pro-mutagen benzo[a]pyrene (B[a]P, 10 µM) did not increase mutation frequency in TK6 cells. In Hepa1c1c7 cells, mutation frequencies varied across exposure groups (50, 50, 29, and 81 per million cells per cell cycle when exposed to 10 µM B[a]P, 5-methylcholanthrene (5-MC), chrysene, or 16,000 µM EMS, respectively). We demonstrate that the choice of cytotoxicity assay and cell line can determine the outcome of the Pig-A mutagenesis assay when assessing a specific mutagen.

## 1. Introduction

Numerous gene mutation assays have been developed to detect mutations. The *Salmonella typhimurium* reverse mutation assay (Ames test), introduced by Bruce Ames in the 1970s, is a rapid and cost-effective tool for assessing the potential mutagenicity (and potential carcinogenicity) of chemicals. This assay involves exposing histidine-requiring strains of *Salmonella typhimurium* to the test agent and observing whether it triggers reverse mutations, thereby restoring histidine biosynthesis. According to current OECD test guidelines, the Ames test also includes strains of *Escherichia coli* that are unable to synthesize tryptophan, broadening the assay’s ability to detect mutagens. A metabolically active fraction of mammalian liver homogenate is required to observe the mutations caused by many chemical compounds, as bacteria lack the necessary enzymes for pro-mutagen activation [1,2]. While the Ames test provides quick results at low cost, its limitations arise from the biological differences between prokaryotic bacteria and eukaryotic human cells, as well as the inherent constraints of the test itself. As a result, compounds identified as mutagenic in this assay may not necessarily induce mutations in humans, necessitating further testing. Substituting the metabolically active fraction with cryopreserved human hepatocytes has addressed some concerns [1]. Additionally, researchers have turned to mammalian cell in vitro tests, with the thymidine kinase (TK) mouse lymphoma assay and the hypoxanthine phosphoribosyl transferase (HPRT) gene mutation assays being the most common [3]. Unlike the Ames test, which detects reversible mutations, the TK and HPRT assays are designed to identify direct mutations in the genes encoding thymidine kinase and hypoxanthine phosphoribosyl transferase, respectively. The TK gene is located on chromosome 11 while the HPRT gene is located on X chromosome, and both are related to the purine nucleotide salvage pathway. In these assays, test chemicals are used to induce mutations, and then the cells are subjected to selective pressure to identify those that have undergone genetic changes. For the HPRT assay, cells with mutations in the HPRT gene are selected using the cytostatic agent 6-thioguanine (6-TG). For the TK assay, mutants are detected using 3-fluorothymidine, which targets cells with mutations in the TK gene [4,5].

Polycyclic aromatic hydrocarbons (PAHs) originate from both anthropogenic and natural sources. They are ubiquitous byproducts of industrial processes, such as the incomplete combustion of fossil fuels, wood, and garbage. Given the extensive use of petroleum and its derivatives in transportation and storage, exposure is inevitable. We evaluated three types of PAHs: benzo[a]pyrene (B[a]P), 5-methylchrysene, and chrysene. Both B[a]P and chrysene are United States Environmental Protection Agency (EPA)-listed PAHs, with B[a]P classified as carcinogenic to humans, and 5-methylchrysene and chrysene considered as probable human carcinogens. B[a]P and the above-mentioned chrysenes are procarcinogens that undergo bioactivation primarily through the diol–epoxide and radical–cationic pathways, leading to DNA adduct formation and potential carcinogenesis. In the diol–epoxide pathway, B[a]P is catalyzed by cytochrome P450 (CYP450) enzymes, especially CYP1A1 and CYP1B1, and epoxide hydrolase into B[a]P-7,8 diol-9,10-epoxide (BPDE) [6]. Research shows that BPDE is the ultimate carcinogen that can covalently bind to DNA, mainly with purine bases forming deoxyguanosine– and deoxyadenosine–DNA adducts [7]. As for the radical–cationic pathway, after catalyzed by CYP or peroxidase, B[a]P generates a radical cation at carbon 6 and covalently binds to DNA [6]. While 5-methylchrysene is not included in the EPA list, previous studies have demonstrated its high carcinogenicity, also requiring bioactivation by CYP450. It is first catalyzed by CYP1A1 into 5-methylchrysene-1,2-diol, and then transformed into the final carcinogen 5-methylchrysene-1,2 diol-3,4-epoxide, which can bind to DNA and trigger G-T transversion mutations [8,9,10]. B[a]P is also known to induce G1/S cell cycle arrest. Following exposure to 3 µM of B[a]P, the proportion of S phase HepG2 cells significantly increased up to 24 h post-exposure, attributed to the upregulation of G1 phase regulators p21 and p27, and downregulation of cyclin D and cyclin E [11,12].

The PIG-A gene encodes the phosphatidylinositol glycan anchor biosynthesis class A (PIG-A) protein, a catalytic subunit of GPI N-acetylglucosaminyltransferase (GPI-GnT) involved in GPI precursor biosynthesis. GPI serves as a plasma membrane anchor for over 150 proteins via the GPI-Aps pathway [13]. The protein is first synthesized in the endoplasmic reticulum (ER) and then attaches to its precursor molecules. The GPI anchor attaches to the protein precursor through an amino bond between the C-terminal of the precursor and the GPI ethanolamine phosphate (EtNP) site. Following this attachment, the GPI-anchored protein (GPI-AP) undergoes a series of remodeling steps in the endoplasmic reticulum (ER) and Golgi apparatus before being transported to the plasma membrane (PM) [13,14]. The mature GPI-AP attaches to the outer leaflet of PM through hydrocarbon chains of PI. PIG-A gene mutation leads to the deficiency of GPI anchors and GPI-Aps on the plasma membrane, such as CD55 and CD59, which can be detected by flow cytometry [15]. PIG-A has emerged as a marker in in vivo research and is used for Paroxysmal Nocturnal Hemoglobinuria (PNH) diagnosis [15,16,17]; however, there is not much information from in vitro studies of this model system. Previous research proved that an increase in the mutation rate of the PIG-A gene is similar to that of an increase in the rate of the HPRT gene in mutator phenotype cancer cells when compared to non-cancer cells [18], demonstrating the potential of the PIG-A gene as a gene mutation marker comparable to HPRT, albeit less complex and time-consuming for detection. In our study, we use flow cytometry as an effective tool to detect PIG-A as a mutated target locus. Phycoerythrin (PE) is a fluorescent dye used to label anti-CD59 antibodies. In this system, the absence of the PE signal indicates functional PIG-A mutations, as these mutations impair the attachment of CD59 to the cell surface.

We assessed the genetic toxicity of B[a]P, chrysene, and 5-methylchrysene on two cell lines: the murine hepatoma cell line (Hepa1c1c7) and the human lymphoblast cell line (TK6). Notably, only the Hepa1c1c7 cell line possesses CYP450 enzymes capable of bioactivating PAHs, whereas TK6 cells lack this ability in theory. One of our objectives was to explore the existence of alternative pathways for PAH-induced carcinogenesis in a cell line that lacked CYP450. As a reference carcinogen for both cell lines, we used ethyl methanesulfonate (EMS), which is a model alkylation agent known to cause gene mutations and chromosomal aberrations. EMS operates via a distinct mechanism from PAHs. It directly binds to DNA, primarily targeting N7-guanine and O6-guanine, with over 99% resulting in G/C-to-A/T transitions [19,20], and induces adducts and DNA damage through alkylation due to its high Swain–Scott constant [21,22]. In this study, the aim is to establish a test system for assessing the genotoxicity of PAHs using the PIG-A gene and flow cytometry.

## 2. Materials and Methods

### 2.1. Materials and Instruments

Both Hepa1c1c7 (CRL-2026) and TK6 cells (CRL-8015) were purchased from the American Type Culture Collection (ATCC, Manassas, VA, USA). RPMI-1640 medium (Lot no. 2687498) and MEM Alpha medium (Lot no. 2472384) were purchased from Gibco, Thermo Fisher (Waltham, MA, USA). Anti-mouse PE-conjugated CD59 antibody (Clone REA287, Lot no. 5230105732), anti-human PE-conjugated CD59 antibody (Clone REA496, Lot no. 1322120604), lyophilized anti-PE MicroBeads (Lot no. 5181221156), LS columns (Lot no. 5180927085), and a QuadroMACS separator were purchased from Miltenyi Biotec (Bergisch Gladbach, Germany). PI (Lot no. 10969300) was bought from Roche Diagnostics Biotech (Mannheim, Germany). Thiazolyl blue tetrazolium bromide (Lot no. MKBL6157V) was purchased from Sigma-Aldrich (St. Louis, MO, USA). Optical density was tested using a Tecan infinite M200 Pro microplate reader (Tecan, Männedorf, Switzerland) and a MACSQuant 10 flow cytometer (Miltenyi Biotec, Bergisch Gladbach, Germany) was used for the fluorescence tests.

### 2.2. Cell Culture

TK6 cells were cultured in complete RPMI-1640, while Hepa1c1c7 cells were cultured in MEM Alpha medium with 10% heated inactivated fetal bovine serum (FBS). TK6 cells were maintained in T75 culture flaks for suspension cells and passaged at a 1:10 ratio every other day to maintain a reasonable cell density. Hepa1c1c7 cells were cultured in T75 flasks treated for adhesive cells and at a 1:10 ratio every three days to keep the cells below 80% confluence. Both cell lines were incubated at 37 °C in an ambient atmosphere supplemented with 5% CO_2_.

### 2.3. Comparison of Cell Viability Assays

Cell counting and viability were measured using flow cytometry and MTT assays. These assays were compared to live and dead cell counting using a hemocytometer and trypan blue exclusion cytotoxicity evaluation as a standard for comparison. When comparing cell-count function, TK6 cells were washed with phosphate-buffered saline (PBS), stained with trypan blue, and counted using a hemocytometer. Viable cells and dead cells that stained blue were summed to give the total and percent viable cell numbers. After cell counting, cells were resuspended to 1 × 10^6^ cells/mL, and 1:10 serial dilutions were used to generate cell concentrations from 1 × 10^6^ to 1 × 10^2^ cells/mL. For both MTT and flow cytometry assays, 100 µL of cells from each concentration were plated in triplicate columns of a transparent 96-well plate with compete medium as the blank control.

The MTT assay was performed following the manufacturer’s protocol. First, 15 mg of thiazolyl blue tetrazolium bromide was fully dissolved in 3 mL of PBS. Then, 10 µL of complete MTT solution was added to each well of the MTT 96-well plate, and cells were incubated with lids in an incubator for 4 hrs. After incubation, 100 µL of MTT solubilization solution was added to each well, and the plate was thoroughly mixed. The plate was read using the Tecan Infinite M200 PRO plate reader at wavelength 570 nm and 690 nm for background correction. The flow cytometer plate was mixed thoroughly before testing, and then immediately loaded onto the flow cytometer. Then, 100 µg/mL PI solution was added to each well at a ratio of 1:100 and tested.

Before counting by hemocytometer, Hepa1c1c7 cells were detached using 0.25% Trypsin-EDTA. The reaction was halted by adding a five-fold volume of complete medium, and the cells were then resuspended and repetitively pipetted to disaggregate any cell clusters. The same staining procedure was used for TK6 cells. A 10-fold dilution gradient from 1 × 10^6^ to 1 × 10^2^ cells/mL was generated. Cells for both methods were cultivated for 20 h before testing. This period of time permitted viable Hepa1c1c7 cells to attach to the surface of the cultureware.

To compare viability function, both cell lines were seeded in a 96-well plate at a concentration of 5000 cells per well with 100 µL of medium. TK6 cells were exposed to 15 µM of B[a]P, and 600 µM of EMS, with 0.5% DMSO and protein-free RPMI-1640 medium as the controls, respectively, for 4 h in nine replications, with three replications for each method. Pure protein-free RPMI-1640 medium was the blank control. Hepa1c1c7 cells were exposed to 15 µM of B[a]P, and 16,000 µM of EMS, with 0.5% DMSO and protein-free MEM Alpha medium as the control for 4 h in nine replications, with three replications for each method. Pure protein-free MEM Alpha medium was the blank control. The detailed exposure procedure is presented below. Cell viability was tested 24 h after exposure. Equations (1) and (2) were used to calculate the % live cells for the MTT assay and PI, respectively.

% live cells of the MTT = (OD_exposure_ − OD_blank_)/(OD_control_ − OD_blank_) × 100 (1)

% live cells of PI = (N_exposure_ /N_control_) × 100 (2)

where OD_exposure_, OD_control_, and OD_blank_ represent the optical density (OD) of the exposure groups, control groups, and blank control, respectively. N_exposure_ and N_control_ represent the live cell number detected by the flow cytometer of the treated and control group.

### 2.4. Antibody Staining

Primary antibody staining was conducted according to the Miltenyi Biotec protocols. TK6 cells were washed twice with PBS buffer, centrifuged at 300× *g* for 5 min, and resuspended in PBS buffer with 0.5% bovine serum albumin (BSA) and 2 mM EDTA at a concentration of 1 × 10^6^ cells per 980 µL of mixed buffer. PE-conjugated anti-human CD 59 antibody (clone: REA496, 20 µL) was added and the mixture was incubated in a refrigerator for 10 min. After incubation, stained cells were washed twice with 10 times the volume of complete medium, and resuspended cells in pre-warmed complete medium.

For Hepa1c1c7 cells, cells were trypsinized, resuspended with complete medium and thoroughly pipetted to break down the clusters. Cells were then resuspended in PBS staining buffer at a concentration of 1 × 10^6^ cells per 450 µL. PE-conjugated anti-mouse CD 59 antibody (clone: REA287, 50 µL) was added to cells and incubated in the refrigerator for 10 min. Cells were washed with 10 times the volume of complete medium after staining.

### 2.5. Generating GPI(+) Cell Populations

**Figure 1:**
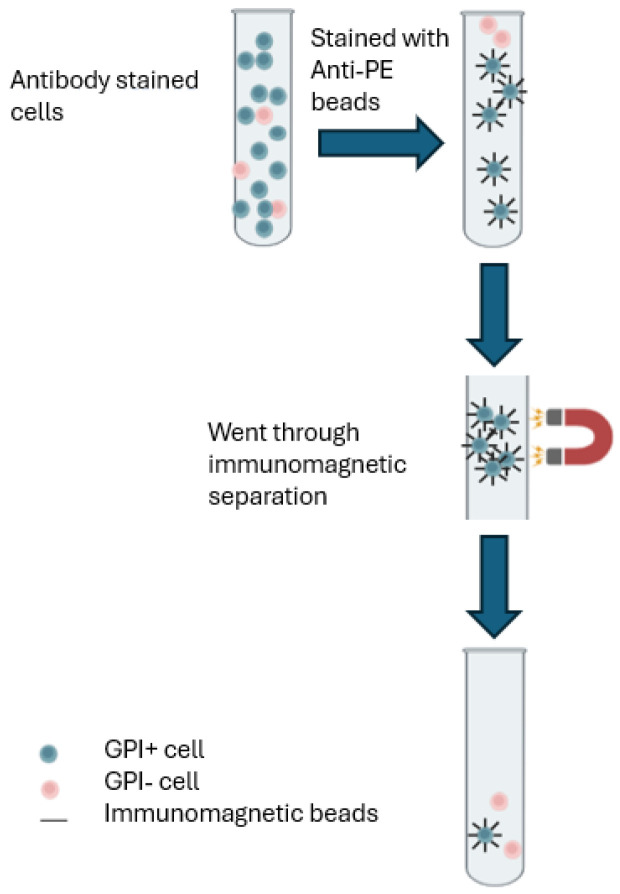
Mutant cell enrichment by separation with anti-CD59 microbeads and paramagnetic separator.

Lyophilized anti-PE microbeads were used for cell separation. Cells were stained with PE-conjugated antibody before separation and the procedure is shown in Figure 1. Then, 1 × 10^6^ cells were centrifuged, resuspended in 80 µL PBS buffer, and mixed with 20 µL of anti-PE microbeads. After being incubated at 4 °C for 15 min, cells were washed with 1 mL PBS buffer and resuspended in 500 µL of PBS buffer. An LS column was placed on the magnetic separator and pre-rinsed with 3 mL PBS buffer before separation. Then, 500 µL cell suspension was applied to the column, and the column was washed three times with 3 mL of PBS buffer. The flow-through containing unlabeled cells (GPI(−) cells) was collected in a sterile 15 mL centrifuge tube. The column was then removed from the separator and 5 mL of PBS buffer was added to flush away the labeled cells. The same procedures were applied to both cell lines and each separation was repeated twice.

### 2.6. Exposure Treatment and Cytotoxicity Experiment

Treatments for both cell lines were performed on day 0 on GPI(+) cell populations. All PAHs were dissolved in dimethylsulfoxide (DMSO). The final concentration for DMSO did not exceed 0.5% for any exposure condition. For the TK6 cell line, 2 × 10^6^ GPI(+) cells were cultured in 10 mL complete RPMI-1640 medium within a T75 flask. Cells were washed and treated with RPMI-1640 protein-free medium containing the test substance (800, 600, 300 µM EMS, or 10 µM B[a]P) in triplicate. For the TK6 cell line, EMS concentrations (800, 600, and 300 µM) were chosen based on a combination of available literature [23,24] and our pilot studies. In our pilot study, cells were exposed to EMS ranging from 100 µM to 800 µM for 4 h and cell survival was assessed 48 h post-exposure. Pure protein-free medium or protein-free medium with 0.5% DMSO were used as controls to compare EMS and B[a]P exposures, respectively. After a 4 h exposure, cells were washed to remove chemical residuals and then cultured in fresh medium within new flasks.

For the Hepa1c1c7 cell line, 2 × 10^6^ GPI(+) cells were seeded in a T75 flask with 5 mL complete MEM Alpha medium 20 h before exposure. After the cells attached, the old medium was washed away, and cells were treated with MEM Alpha protein-free medium containing the test substance (10 µM B[a]P, 10 µM 5-MC, 10 µM chrysene, and 16,000 µM EMS) in triplicate. For the Hepa1c1c7 cell line, limited information on EMS dosing was available in the literature, so we conducted a pilot study. Cells were exposed to EMS concentrations ranging from 500 µM to 16,000 µM for 4 h. Cell survival was then assessed at 48 h after exposure, and we found that only the 16,000 µM EMS concentration caused a significant reduction in survival, leaving 10% of the cells viable. Based on these results, 16,000 µM EMS was selected to ensure sufficient cytotoxic impact for mutagenicity testing while maintaining adequate cell viability for further analysis. Pure protein-free medium or 0.5% DMSO protein-free medium were used as control groups for EMS and PAH exposures, respectively. After exposure for 4 h, cells were washed, trypsinized, and cultured in new T75 flasks.

For both cell lines, 2000 cells for each treated and control group were transferred into a 96-well plate in 100 µL medium per well. Cytotoxicity was tested every other day from day 2 to day 8 after exposure using flow cytometry.

Relative Increase in Cell Counts (RICCs) and Relative Population Doubling (RPD) are commonly utilized indices serve as indicators for cytotoxic effects [23]. The RICC was calculated from day 4 to day 8 using Equation (3).

RICC = (N_treated_ − 2000)/(N_control_ − 2000) ∗ 100 (3)

N_treated_ and N_control_ represent the cell numbers in the treated group and control group, respectively. Relative Population Doubling (RPD) was calculated with Equation (4).

RPD = (No. of population doublings in treated group/No. of population doubling in control group) ∗ 100(4)

### 2.7. Pig-A Gene Mutation Assay

For both cell lines, treated cells were cultured in T75 flasks and passage every 2 days to prevent overgrowth of cultures. The PIG-A mutagenicity test was conducted on day 10. On day 10, cells were stained with PE-conjugated CD-59 antibody before testing and analyzed using the flow cytometer.

Mutation frequency with the RICC to assess cytotoxicity effects was calculated with Equation (5).

Mutation Frequency = ((N − N_0_)/(n ∗ RICC)) ∗ 100 (5)

Here, N represents the number of mutated cells detected by flow cytometer, N_0_ denotes the GPI(−) background before exposure, and n represents the number of potential cell cycles over the 10-day period.

Mutation frequency with RPD to assess cytotoxicity effects was calculated with Equation (6).

Mutation frequency = ((N − N_0_)/(n ∗ RPD)) ∗ 100 (6)

Here, N represents the number of mutated cells tested on day 10, N_0_ denotes the GPI(−) background, and n represents the number of cell cycles for the group.

Cell cycle numbers (n) for all groups were estimated based on the cytotoxicity experiment, with calculations performed every two days. Since cell count began on day 2, the total proliferation period was 8 days. If the cell number was lower than in the previous test, the cell cycle number for those two days was considered 0. For groups exhibiting the same growth rate as the control group (e.g., B[a]P, DMSO, and medium groups for the TK6 cell line; chrysene, DMSO, and medium control for the Hepa1c1c7 cell line), the cell cycle number was calculated using Equation (7), as shown below:

Cell cycle number = (8 × 24)/t(7)

Here, t represents the doubling time (16 h per cell cycle for TK6 cells and 18.1 h per cell cycle for Hepa1c1c7 cells). For groups with different growth rates, Equation (8) was utilized, which is shown below.

Cell cycle number = (N_x+2_/N_x_) ^½^ (8)

N_x_ and N_x+2_ represent the cell numbers tested by the cytotoxicity number.

### 2.8. Statistical Analysis

Statistical differences between the control and treated samples were evaluated using one-way analysis of variance (ANOVA), followed by Tukey’s Honest Significant Difference (HSD) test for post hoc analysis.

## 3. Results

### 3.1. Comparison of Cell Count Methods

**Figure 2:**
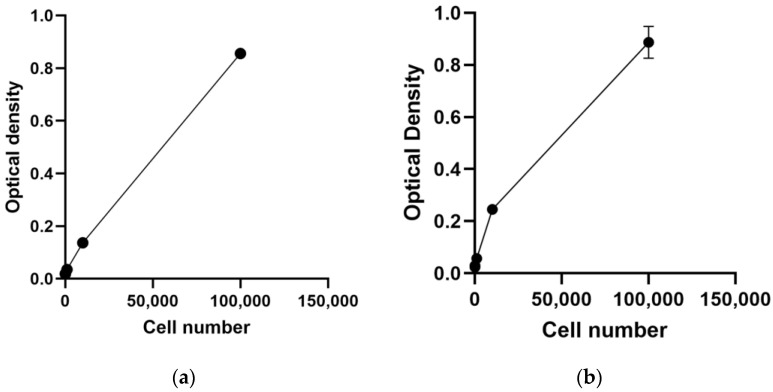
Results for the MTT assay cell-count function: (**a**) represents TK6 cell line and (**b**) represents the result of Hepa1c1c7 cell line.

Cytotoxicity assessment is pivotal in genetic toxicology experiments for evaluating the impact of chemical exposure. Table 1 and Figure 2 present the results of the MTT assay cell-count function for both cell lines.

As depicted in Table 1, the MTT assay demonstrates a detection limit in both cell lines. Notably, in the TK6 cell line, there is no significant difference observed between the 1 × 10^2^ cells/mL and blank groups, indicating that the MTT assay fails to detect cell numbers lower than 10. Conversely, the MTT assay exhibits a higher limit of detection (LOD) for the Hepa1c1c7 cell line (100 cells per well) compared to the TK6 cell line

OD is supposed to be linear related to cell number; however, the linear relationship in the Hepa1c1c7 cell line, especially with a high cell density, is not as strong as in TK6 cell line. In Figure 2b, a marked decrease in the OD slope is evident after the 1 × 10^4^ cells per well group of the Hepa1c1c7 cell line, possibly due to cell saturation. Previous studies have reported a plateau level in OD with increasing cell density, albeit with variations in concentration limits across different cell types [24]. Our experiment suggests the necessity for additional groups above 1 × 10^5^ and between 1 × 10^4^ and 1 × 10^5^ per well for further research.

**Figure 3:**
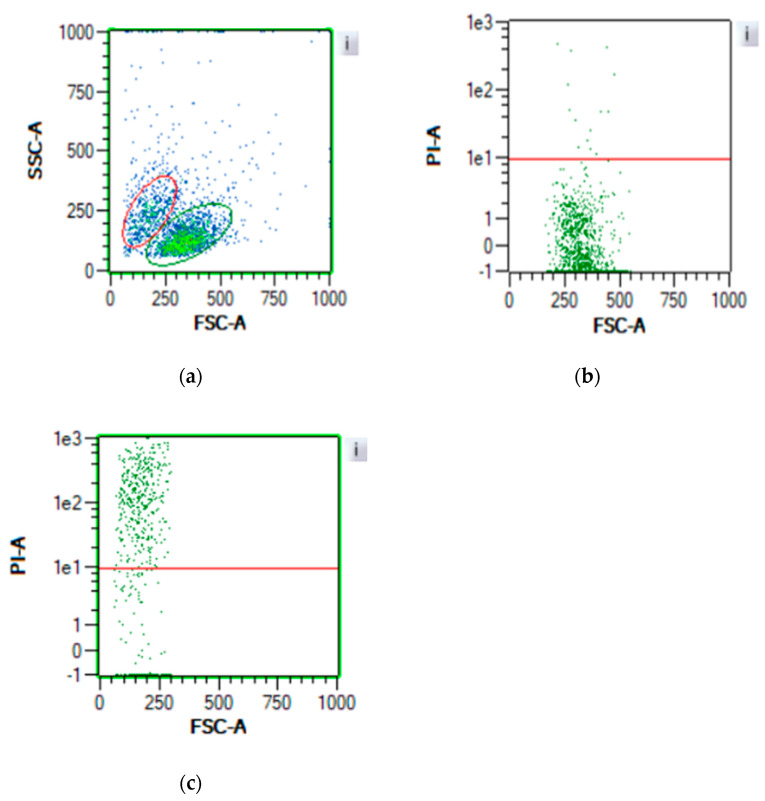
Gating strategy for TK6 cell line: (**a**) cell selection through FSC and SSC channel; (**b**) PI signal of green population in (**a**); (**c**) PI signal of red population in a. Cells in the red region are mostly dead, which indicates that TK6 cells go through a consistent morphology change after death.

**Figure 4:**
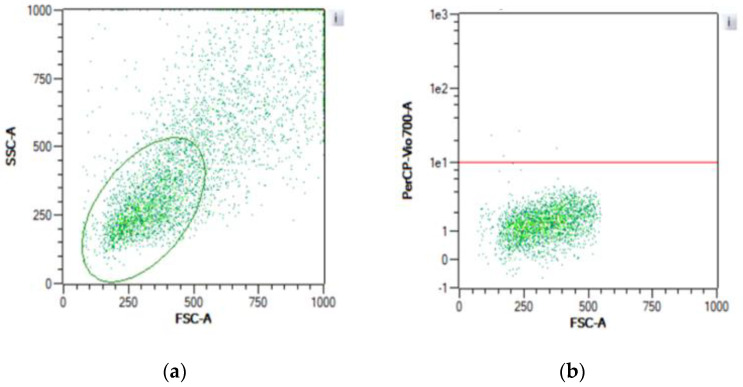
Gating strategy for Hepa1c1c7 cell line: (**a**) cell selection through FSC and SSC channels, which represent cell dimension; (**b**) live cell selection with a low PI signal. PI cannot travel through intact cell membranes and membranes of dead cells lose their integrity.

Figure 3 and Figure 4 depict the gating strategy utilized for flow cytometry analysis of cell viability. In Figure 3a, the flow cytometer identifies two distinct populations, delineated by the red and green regions. Following staining with PI, cells within the green region exhibit a low PI signal (Figure 3b), while those within the red region display a high PI signal (Figure 3c). This observation underscores the morphological changes experienced by TK6 cells post-mortem. Maintaining plasma membrane integrity is crucial for cellular viability, and PI serves as a fluorescent indicator of cell membrane permeability due to its high hydrophobicity. Although prolonged exposure to PI may induce cytotoxic effects, short-term exposure or its use as an endpoint assay is recommended [25].

**Figure 5:**
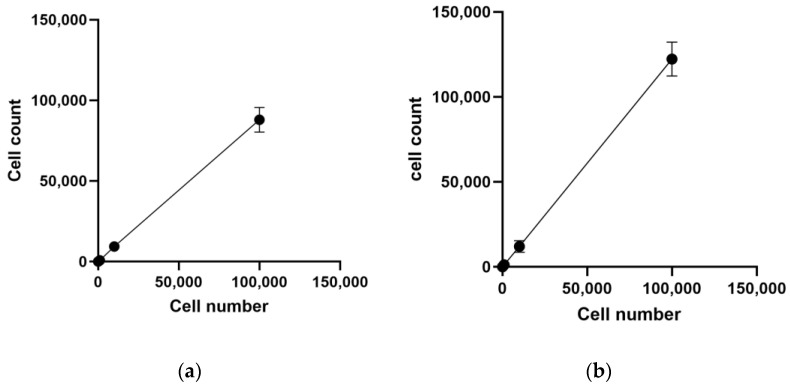
Results for the flow cytometer cell-count function. The Y-axis represents cell number counted by flow cytometry: (**a**) TK6 cell line cell count; (**b**) Hepa1c1c7 cell line cell count.

Table 2 and Figure 5 present the outcomes of flow cytometry analysis using PI. Notably, a robust linear relationship is observed between flow cytometry and hemocytometry techniques. Both PI and trypan blue serve as assays for assessing plasma membrane permeability, with the primary distinction being that PI binds to double-stranded DNA and emits fluorescence upon activation.

**Figure 6:**
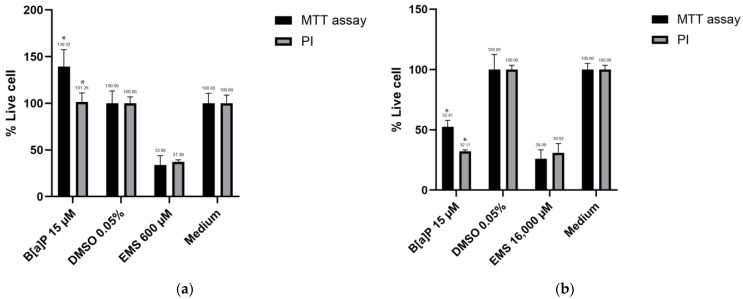
Results for the comparison of cell viability function for the MTT assay and PI: (**a**) cell viability result for TK6 cell line; (**b**) cell viability result for Hepa1c1c7 cell line. Statistical analysis was performed using one-way ANOVA followed by Tukey’s Honest Significant Difference (HSD) test. Groups marked with * are significantly different from the control group (* *p* < 0.05). Error bars represent the standard deviation from the mean for triplicate experiments.

The application of the MTT assay is constrained within a specific cell number range, from 100 to 1 × 10^5^ cells for the TK6 cell line and from 1 × 10^3^ to 1 × 10^4^ cells for Hepa1c1c7 per well. To accommodate this limitation, both cell lines were seeded in two 96-well plates at a concentration of 5 × 10^3^ cells per well. As the cell number was too low, a hemocytometer was not utilized as a control. Figure 6 illustrates the results obtained.

Interestingly, exposure to B[a]P did not induce any detectable cytotoxic effects on the TK6 cell line, yet the MTT assay yielded exceedingly high results. This discrepancy may be attributed to the high hydrophobicity of B[a]P, leading to increased light absorbance by residual B[a]P present in the well. A similar trend was observed in the Hepa1c1c7 cell line. Notably, the live cell percentage determined by the MTT assay post-exposure to B[a]P was 20% higher compared to that obtained via the PI method for the Hepa1c1c7 cell line.

One potential source of residual B[a]P post-exposure could be attributed to B[a]P attachment to the microplate. Previous studies have demonstrated the binding of B[a]P to polystyrene, the material commonly used in microplate production [26,27,28,29]. In our experimental setup, cell transfer to new plates post-exposure was performed to mitigate the influence of residual B[a]P binding to the microplates. Alternatively, the source of B[a]P could be its binding to the cells themselves.

Overall, the MTT assay proved to be time-consuming and less consistent compared to PI staining coupled with flow cytometry for both cell counting and viability assessment. Consequently, the latter method was employed for subsequent research endeavors.

### 3.2. Cytotoxicity Assessment Post-Exposure

**Figure 7:**
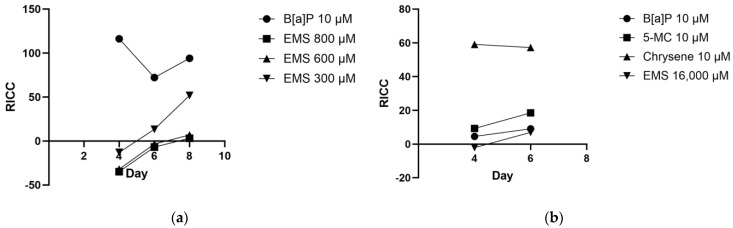
RICC for all exposure groups compared to controls in both cell lines: (**a**) RICC for TK6 cell line; (**b**) RICC for Hepa1c1c7 cell line.

In our study, cell viability was assessed every other day from day 2 to day 8 post-exposure to the test substances. Table 3 and Table 4 illustrate the cell viability of TK6 and Hepa1c1c7 cells over time, respectively, and Figure 7 shows the RICC for both cell lines. The lowest positive RICC point was utilized for subsequent mutation frequency analysis.

The normal doubling time for TK6 cells is approximately 16 h per cell cycle. Notably, the viable cell number decreased in all groups from day 0 to day 2, including the control groups, indicating potential cell loss during processes such as centrifugation and cell washing. Although OECD guidelines recommend centrifugation at 230× *g* for 5 min, we applied 5 min of 300× *g* centrifugation in our experiment [30]. Another factor contributing to cell loss may be the use of phosphate-buffered saline (PBS) buffer, as reported by Chen et al. (2002) [4], where cell samples treated with PBS exhibited significantly lower viability compared to the control group [31]. However, the exact cause of cell loss due to the process remains unknown.

On day 4, the RICC for all EMS exposure groups was negative due to lower live cell numbers compared to day 0. However, by day 6, the RICC for the EMS 300 µM group became positive, while the other two EMS groups did not reach 2000 cells per well until day 8. Additionally, the doubling time for TK6 cells was prolonged to 21 h per cell cycle after EMS exposure.

After exposure, the RICCs for EMS 800 µM (RICC_low_ = 3.16) and 600 µM groups (RICC_low_ = 6.74) were relatively low compared to the findings of Krüger et al. (2015) [15] with 800 µM EMS (RICC_low_ = 10) under the same exposure period. On day 6, a significant difference in live cell numbers between the B[a]P and DMSO groups was observed on day 6. B[a]P is a procarcinogen and needs to be bioactivated by CYP450 and exposure to B[a]P induces cell apoptosis [12]. Despite the knowledge that TK6 cells cannot bioactivate B[a]P, the reason for the detected difference in live cell numbers remains unknown.

The cell viability of Hepa1c1c7 cells is summarized in Table 4. The normal doubling time for Hepa1c1c7 cells is approximately 18.1 h. Notably, after day 6, both control groups experienced overgrowth, leading to a decrease in cell numbers by day 8. To assess RICC, the values for all exposure groups from day 4 to day 8 were compared, and the lowest positive RICC for each group was utilized for further analysis.

Upon analyzing the exposure to polycyclic aromatic hydrocarbons (PAHs), both the B[a]P and 5-MC groups exhibited a substantial cytotoxic effect. Specifically, on day 2, the B[a]P and 5-MC groups displayed lower cell numbers compared to the DMSO group. Cells exposed to B[a]P (doubling time of 26.1 h per cell cycle) and 5-MC (doubling time of 25.1 h per cell cycle) demonstrated an extended doubling time. In contrast, the growth rate for the chrysene group remained comparable to that of the control group (18.1 h per cell cycle). Additionally, in comparison to TK6 cells, Hepa1c1c7 cells exhibited lower sensitivity to EMS.

### 3.3. Pig-A Gene Mutation Assay

**Figure 8:**
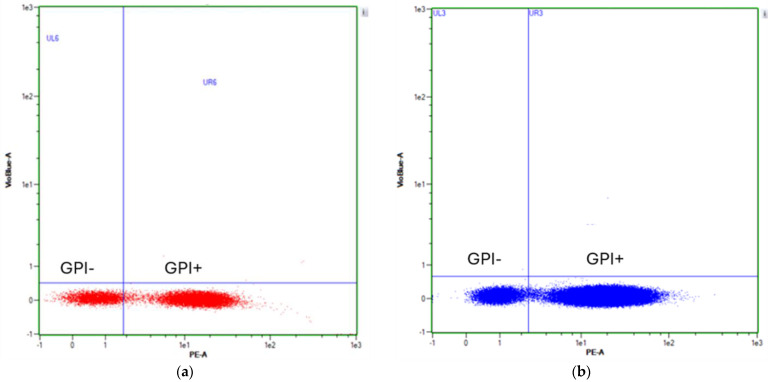
PIG-A gating strategy: (**a**) gating strategies for separating GPI(−) (lower left region) and GPI(+) (lower right region) of TK6 cell line; (**b**) gating strategies for separating GPI(−) (lower left region) and GPI(+) (lower right region) of Hepa1c1c7 cell line. PIG-A gene mutated cells were not stained with anti-CD59 antibodies and had no PE signal that was conjugated with antibodies.

**Figure 9:**
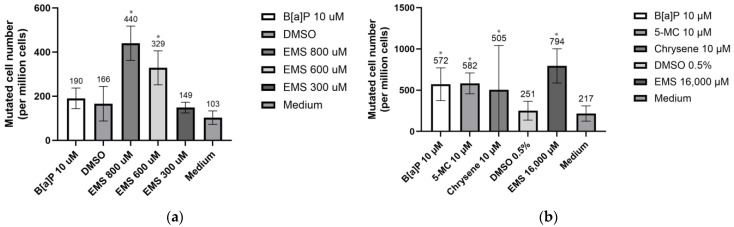
GPI(−) cell number detected by flow cytometry: (**a**) GPI(−) cell number in TK6 cell line; (**b**) GPI(−) cell number in Hepa1c1c7 cell line. Statistical analysis was performed using one-way ANOVA followed by Tukey’s Honest Significant Difference (HSD) test. Groups marked with * are significantly different from the control group (* *p* < 0.05). Error bars represent the standard deviation from the mean for triplicate experiments.

Gate locations for PIG-A gene separation on flow cytometry were based on testing the PE signal of mixed GPI(+) and GPI(−) cells with anti-CD59 antibody staining (Figure 8). After cleansing, the GPI(−) background in TK6 and Hepa1c1c7 cells was 0.01% and 0.02%, respectively. The GPI(−) background is relatively high when compared to previous studies and one of the reasons might be the efficiency of the microbeads and magnetic separator. Others were labeled with two GPI-anchored proteins (CD59 and CD55) when generating GPI(+) populations in the TK6 cell line [15,32]. The mutated cell number on day 8 detected by Pig-A Gene Mutation Assay is presented in Figure 9. There was no statistically significant difference between the two control groups in either of the cell lines, indicating that 0.5% DMSO does not increase mutation frequency and shows no evidence genetic toxicity [33]. In the TK6 cell line, among all the exposure groups, EMS 800 µM (440 out of a million cells) and 600 µM (329 out of a million cells) groups showed significantly more GPI(−) cells when compared with medium control groups (103 out of 1,000,000 cells). The GPI(−) cell number in the EMS 300 µM group is not significantly different from that of medium control, indicating 4 h exposure to 300 µM EMS has no mutation effect on TK6. Krüger et al. (2015) [15] discovered an increased TK6 GPI(−) frequency after being exposed to 200 µM EMS for 4 h. Zhou et al. (2021) [32] also observed the genotoxicity of TK6 cells following exposure to 200 µM EMS. These discrepancies suggest potential differences in experimental conditions or sensitivity between studies.

**Figure 10:**
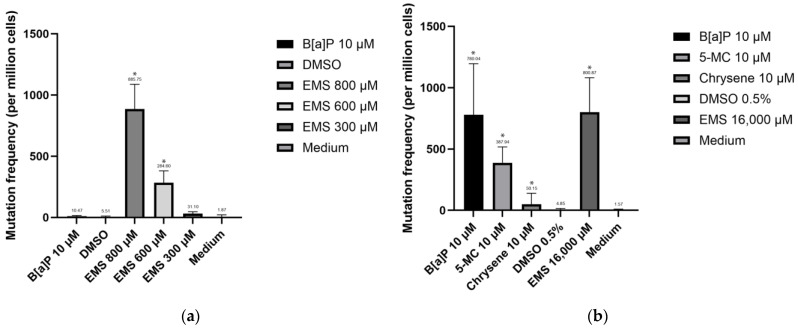
Mutation frequency for cell lines RICC: (**a**) TK6 cell line mutation frequency; (**b**) Hepa1c1c7 cell line mutation frequency. Statistical analysis was performed using one-way ANOVA followed by Tukey’s Honest Significant Difference (HSD) test. Groups marked with * are significantly different from the control group (* *p* < 0.05). Error bars represent the standard deviation from the mean for triplicate experiments.

The results are presented in Figure 10. For the TK6 cell line, a 4 h exposure to 800 µM EMS increased the mutation frequency to 886 ± 201.61 per million cells per cell cycle, while exposure to 600 µM EMS over the same period raised the frequency to 285 ± 95.85 per million cells per cell cycle. Conversely, the EMS 300 µM group showed a frequency of 32 ± 15.48 per million cells per cell cycle, which was not statistically significant compared to controls. The spontaneous mutation frequency observed for the TK6 cell line in our experiment was 2 ± 19.50 per million cells per cell cycle, exhibiting significantly higher variation compared to the findings of Krüger et al. in 2015 [15] (0.76 ± 0.43 per million cells per cell cycle). Notably, no significant difference was observed between the B[a]P, DMSO, and medium control groups, indicating that as expected, B[a]P did not exhibit genetic toxicity on TK6 cells, consistent with the background knowledge that TK6 cells lack CYP450 isoforms and cannot bioactivate B[a]P to its carcinogenic form, BPDE.

In the Hepa1c1c7 cell line, mutation frequencies for the DMSO and medium control groups did not show statistically significant differences. The spontaneous frequency for Hepa1c1c7 cells was 2 ± 8.73 per million cells per cell cycle. However, all treated groups exhibited significantly higher mutation frequencies compared to the control groups. Exposure to B[a]P and 5-MC increased the mutation frequency to 781 ± 415.87 and 388 ± 128.83 per million cells per cell cycle, respectively. EMS exposure resulted in a mutation frequency of 801 ± 280.81 per million cells per cell cycle. Interestingly, the chrysene group exhibited an exceptionally high mutation frequency at replication 6 (229 per million cells per cell cycle). It is noteworthy that mutations can occur throughout all stages of the experiment, and the variation in mutation frequency may stem from replications with cells mutated at earlier stages possessing more mutated cells by day 10.

However, one limitation of using RICC values on day 2 for each group resulted in negative values that prevented their use in calculations. This is attributed to the observed decrease in cell numbers from day 2 to day 10, despite each group initially containing the same number of cells (2 × 10^3^ cells per well). The extent of reduction in cell numbers varied among groups, resulting in different baseline cell counts at the onset of cell growth. Consequently, we attempted to calculate the Relative Population Doubling (RPD)

**Figure 11:**
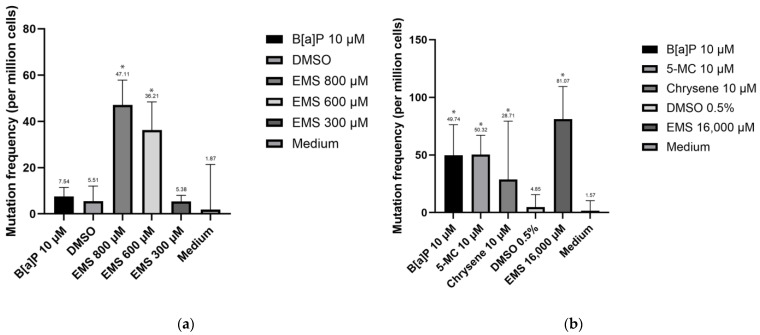
Mutation frequency for cell lines corrected for RPD: (**a**) TK6 cell line frequency; (**b**) Hepa1c1c7 cell line mutation frequency. Statistical analysis was performed using one-way ANOVA followed by Tukey’s Honest Significant Difference (HSD) test. Groups marked with * are significantly different from the control group (* *p* < 0.05). Error bars represent the standard deviation from the mean for triplicate experiments.

The calculated cell cycle numbers are presented in Table 5. Subsequently, the final mutation frequency was determined by dividing the increased number of mutated cells from the background by the cell cycle number. The results are depicted in Figure 11. Notably, the mutation frequency for both cell lines experienced a drastic decrease. This decline can be primarily attributed to the reduction in cell numbers after exposure, resulting in varying baseline cell counts at the initiation of cell growth. This indicates that RICC overestimated the cytotoxicity effect.

## 4. Discussion

Our objectives were (a) to establish an in vitro PIG-A gene mutation assay for PAHs using flow cytometry, (b) to investigate different data normalization and analysis techniques, and (c) to investigate the ability of the in vitro system to probe test chemicals for differences in potency compared to an index chemical (EMS).

Despite concerns about trypsinization affecting membrane protein levels (e.g., surface proteins CD55 and CD59 used for the PIG-A assay), the literature suggests mild trypsinization does not significantly impact this parameter [34,35]

### 4.1. Cell Viability Test Comparison

The common definition of cytotoxicity encompasses cell death, but it can also refer to non-lethal adverse effects on cells. Cytotoxicity is a widely used measurement in in vitro tests because it provides insights into the interaction between cells and tested chemicals, aiding in the establishment of exposure gradients for further research. Moreover, when cell death is not the primary endpoint, cytotoxicity becomes a potential confounder that needs to be carefully considered [23]. There are four main types of viability test assay for in vitro experiments: (1) non-invasive structural cell damage assays; (2) invasive structural cell damage; (3) cell growth test; (4) cellular metabolism.

We compared the MTT assay and PI staining using flow cytometry for cell viability assessment, focusing on cell count and viability functions, while using trypan blue in microscopy as a standard. Common challenges encountered when utilizing trypan blue in microscopy include subjective judgments regarding stained cells and debris, potential dye absorption by live cells through diffusion during prolonged exposure, and operator-dependent inconsistencies [25,36,37,38]. Moreover, both PI and trypan blue assays have been reported to exhibit cytotoxic effects with prolonged exposure. This should not be a problem as samples were tested immediately after stained with PI or trypan blue.

The MTT assay evaluates cell metabolism and is susceptible to alterations in the culture environment, such as contact inhibition of adherent cells nearing confluency [25,39]. Moreover, factors including incubation period, temperature, and protein content in complete medium can influence MTT assay outcomes [40,41]. Phenol red in complete medium can interfere with light absorbance, while acidification of the solubilizing solution aids in color change to yellow, reducing interference [25,41]. These factors contribute to the observed low consistency of the MTT assay [24]. Furthermore, the MTT itself exhibits cytotoxic effects on cells, as formazan crystals penetrate the cell membrane, leading to cell death [25,40,42]. Consequently, the MTT assay tends to underestimate cell viability.

The MTT assay demonstrated a plateau for Hepa1c1c7 cells at 1 × 10^4^ to 1 × 10^5^ cells per well, consistent with previous findings [24]. This plateau likely arises from contact inhibition in adherent cells, leading to reduced metabolic activity upon reaching confluency. In contrast, TK6 cells did not exhibit saturation up to 1 × 10^6^ cells per ml, which is typically the upper limit for TK6 concentrations. Compared to PI staining, the MTT assay exhibited less consistency and susceptibility to various factors such as incubation period, cell number, and environmental conditions, as well as potential cytotoxicity [40]. The MTT assay’s reliance on the reduction in MTT and formation of formazan may lead to false positives due to its reaction with reductive components in the medium [42]. Although PI staining may induce cytotoxicity with prolonged exposure, this was mitigated by immediate testing post-staining in our experiment. PI staining can also be influenced by extracellular nucleic acids (e.g., eDNA), which are signals of oxidative stress and mainly derived from dead cells [37,43]. Cell-free DNA (cfDNA) or eDNA was reported to be the signal of oxidative stress in human cells [44,45] and it was detected in the medium of intact cells, where the main source of eDNA was dead cells [44]. Notably, flow cytometry measures viability based on both PI signal and cell size, allowing exclusion of small cell debris (Figure 3a and Figure 4a).

Another issue observed in our experiment was the MTT assay’s overestimation of viability following exposure to B[a]P (15 µM) in both cell lines. B[a]P’s hydrophobicity and potential autofluorescence may lead to increased optical density readings. To improve future research, we suggest narrowing the interval between groups in the MTT assay to establish a standard curve and conducting additional experiments to determine the saturation point for Hepa1c1c7 cells.

### 4.2. Cytotoxicity Experiments

Cell viability assessment is critical for in vitro toxicology studies, necessitating appropriate cytotoxicity measurement methods. Commonly used indicators include RPD and RICC, with RPD and RICC being preferred in the absence of cytochalasin B [46,47]. In previous studies, research groups have often selected the lowest relative increase in cell count (RICC) point throughout longitudinal cytotoxicity tests as the index of cytotoxicity for treated groups [15,32]. However, in our research, we evaluated the cytotoxicity test effect using two methods to determine which one fit our experimental context the best. A statistically significant difference between RICC and RPD was detected when assessing cell viability. This discrepancy arises because the cell numbers decreased in almost all groups to different extents, leading to varying baseline cell numbers when cells recovered and began growing. This repeated cell decrease indicates it was not an artifact but a process that cells underwent post-exposure. This finding aligns with previous research indicating that RICC values are influenced by initial numbers of treated cells [46].

RPD can mitigate the influence of initial cell number inequity since cell cycle numbers were counted directly from the cytotoxicity test. However, RPD also has limitations. When the cell numbers decreased, the cell cycle number was counted as 0, which is not accurate. Cells were still growing but more were dead by apoptosis. Another limitation is the potential overgrowth of control groups in cytotoxicity tests.

To address these limitations, we took several steps in our PIG-A gene mutation assay. Firstly, cells were passaged every other day to prevent overgrowth, and cell cycle numbers for control groups were calculated based on cell doubling time. Additionally, the cytotoxicity test was conducted for only 8 days, and for treated groups not recovered by day 8 (such as all three EMS exposure groups in the TK6 cell line), an estimation of the cell cycle number was made based on the cell cycle number from day 6 to 8. While limited information on EMS exposure in Hepa1c1c7 cells was available, our experiment indicated that Hepa1c1c7 cells are 20-fold less sensitive to the genotoxic effect of EMS than TK6 cells. This result indicates that the effects of chemicals are tissue dependent. Mechanistic studies are needed to determine the basis for the differences in EMS cytotoxicity and mutagenesis between these cell lines.

Regarding B[a]P exposure, we observed no cytotoxicity effect on TK6 cells, consistent with the knowledge that B[a]P is a procarcinogen requiring bioactivation by CYP450 enzymes not expressed in TK6 cells. B[a]P’s cytotoxicity is mediated through apoptosis, as evidenced by increased p53 accumulation in Hepa1c1c7 cells [48]. One suggestion for improvement involves extending the cytotoxicity test to 10 days to better estimate RPD, particularly for treated groups. This extended duration would provide a more comprehensive assessment of cytotoxic effects.

### 4.3. Pig-A Gene Mutation Assay

The PIG-A gene mutation assay is based on the deficiency of the glycosylphosphatidylinositol (GPI) anchor, which is crucial for anchoring proteins to the cell membrane. Among the 26 genes regulating the GPI anchor, PIG-A is unique as it is located on the X chromosome, resulting in a single functional copy of the gene in either male or female cells. Mutations in PIG-A lead to a deficiency in GPI anchor synthesis, affecting the anchoring of proteins such as CD55, CD59, and CD24 to the cell membrane [17,49,50]. In our experiment, we focused on CD59 as the GPI-anchored protein, while other studies have investigated both CD59 and CD55 in TK6 cell lines. This discrepancy may contribute to the relatively high GPI(−) background after cleansing observed in both cell lines compared to previous studies. EMS is a well-known direct alkylating agent that induces DNA damage by forming DNA adducts, primarily targeting N7-guanine and O6-guanine, leading to G/C-to-A/T transitions. Many polycyclic aromatic hydrocarbons (PAHs) are procarcinogens requiring bioactivation before exerting their genotoxic effects. They can undergo two main pathways: the formation of diol epoxides metabolites, which bind to DNA forming adducts after bioactivation by CYP450 enzymes, or the radical–cationic pathway [6,8,9,10,19,20,51]. Mutation frequencies for both cell lines are presented in Figure 9. In the TK6 cell line, the spontaneous mutation frequency on the PIG-A gene is similar to previous studies, but the variance is higher in our study, potentially due to differences in the timing of mutations. Cells that mutated at an early stage proliferated more, resulting in a higher final number of mutated cells on day 10. Another potential reason for the variance is the method used when generating GPI(+) cell populations.

When evaluating the genetic toxicity effects of chemicals, the results vary depending on the cytotoxicity assessment method used. In the TK6 cell line, using either Relative Increase in Cell Count (RICC) or Relative Population Doubling (RPD), EMS exposure at 800 µM and 600 µM significantly increased the apparent mutation frequency, while no significant difference was observed between the EMS 300 µM group and the control group. Similarly, in the Hepa1c1c7 cell line, mutation frequencies for all exposure groups were significantly higher than those of control groups using both cytotoxicity measurements.

However, the mutation frequencies calculated with RICC for all exposure groups are higher than those with RPD. For instance, with RICC, the mutation frequency of the Hepa1c1c7 cell line increased 159.83 times after exposure to B[a]P (781 ± 415.87 per million cells per cell cycle), and the genetic toxicity effect of B[a]P was 15.55 times that of chrysene (1 ± 88.52 per million cells per cell cycle), which tracks well with the animal-derived relative potency factor (RPF) for chrysene. Using RPD, the mutation frequency of the Hepa1c1c7 cell line increased 10.26 times after exposure to B[a]P (50 ± 26.52 per million cells per cell cycle), and the genetic toxicity effect of B[a]P was 1.73 times that of chrysene (29 ± 50.68 per million cells per cell cycle). This indicates that the results vary with the cytotoxicity measurement used, and RICC can amplify the differences in the genetic toxicity effects between chemicals.

While there is limited information about in vitro PIG-A gene mutation assays on the Hepa1c1c7 cell line, our research demonstrates that using the Hepa1c1c7 cell line in this assay is practical. Our research confirms that the in vitro PIG-A gene mutation assay is a useful method for testing potential carcinogens such as PAHs and generates a quantitative method for PAH genetic toxicity comparison with mutation frequency. We also compare the data normalizations with RICC and RPD. However, there are limitations to this method. The flow cytometer can only detect phenotype changes after mutation and cannot identify silent mutations [52] or mutations associated with epigenetic changes [53]. Combining the PIG-A mutation assay with whole-genome sequencing may enhance our ability to detect every change in the DNA sequence, providing a more comprehensive understanding of mutagenic effects. Despite its economic and time-efficient nature, further improvement of the assay through complementary techniques is warranted.

## Figures and Tables

**Table 1:** Cell count result of MTT assay for both TK6 and Hepa1c1c7 cells.

Cell Number (per Well)	TK6 Cells		Hepa1c1c7 Cells	
	Optical Density	Standard Deviation	Optical Density	Standard Deviation
1 × 10^5^	0.8555	0.0202	0.8874	0.0608
1 × 10^4^	0.1366	0.0207	0.2455	0.0094
1 × 10^3^	0.0350	0.0005	0.0567	0.0032
1 × 10^2^	0.0194	0.0003	0.0296 **	0.0004
10	0.0180 *	0.0004	0.0272 **	0.0012
0	0.0174 *	0.0004	0.0241 **	0.0009

* represents TK6 groups with no statistically significant difference on OD (*p* < 0.05); ** represents Hepa1c1c7 groups with no statistically significant difference on OD (*p* < 0.05).

**Table 2:** Cell count result of flow cytometry for both TK6 and Hepa1c1c7 cells.

Cell Number (per Well)	TK6 Cells		Hepa1c1c7 Cells	
	Flow Cytometry	Standard Deviation	Flow Cytometry	Standard Deviation
1 × 10^5^	88,000	7598	122,333	88,000
1 × 10^4^	9400	1585	12,040	9400
1 × 10^3^	789	176	1179	789
1 × 10^2^	88	10	101	88
10	4	2	6	4
0	0	0	1	0

**Table 3:** Live cell numbers per well for TK6 cells after treatment through day 0 to 8.

Day	B[a]P 10 µM		DMSO 0.5%		EMS 800 µM		EMS 600 µM		EMS 300 µM		Medium	
	Mean	SD	Mean	SD	Mean	SD	Mean	SD	Mean	SD	Mean	SD
0	2000	0	2000	0	2000	0	2000	0	2000	0	2000	0
2	1131	245.06	979	125.13	207	81.11	289	35.18	462	74.69	901	125.09
4	8212	2506.52	7346	759.72	90	23.00	250	90.94	1292	168.48	7481	1198.09
6	22,383	3295.03	30,262	3078.27	525	259.66	1327	252.14	4644	564.57	21,897	3630.51
8	52,942	3389.86	56,195	6722.58	2985	1485.97	5372	670.39	27,962	4063.47	52,026	3498.02

**Table 4:** Live cell number for Hepa1c1c7 cells through day 0 to 8.

Day	B[a]P 10 µM		5-MC 10 µM		Chrysene 10 µM		DMSO 0.5%		EMS 16,000 µM		Medium	
	Mean	SD	Mean	SD	Mean	SD	Mean	SD	Mean	SD	Mean	SD
0	2000	0	2000	0	2000	0	2000	0	2000	0	2000	0
2	781	267.30	892	229.17	1563	679.44	2186	308.45	614	106.91	2230	440.23
4	2627	426.97	3287	443.34	10,202	4773.88	15,884	1719.79	1710	349.57	15,784	3374.19
6	6840	1185.97	11,872	2649.98	32,600	10,903.44	55,434	9388.86	5154	1001.52	47,334	6674.03
8	27,817	5782.53	37,750	5379.13	53,880	7568.82	49,550	7280.04	19,834	1505.55	45,484	6653.85

**Table 5:** Cell cycle number for all groups in two cell lines.

TK6		Hepa1c1c7	
Chemical	Mean	Chemical	Mean
B[a]P 10 μM	12	B[a]P 10 μM	7.48
DMSO 0.5%	12	5-MC 10 μM	7.60
EMS 800 μM	7.22	Chrysene 10 μM	10.61
EMS 600 μM	6.32	DMSO 0.5%	10.61
EMS 300 μM	9.02	EMS 16,000 μM	7.33
Medium	12	Medium	10.61

## Data Availability

The data presented in this study are available in article.
